# The functional interplay of *Helicobacter pylori* factors with gastric epithelial cells induces a multi-step process in pathogenesis

**DOI:** 10.1186/1478-811X-11-77

**Published:** 2013-10-07

**Authors:** Gernot Posselt, Steffen Backert, Silja Wessler

**Affiliations:** 1 Division of Molecular Biology, Department of Microbiology, Paris-Lodron University, Salzburg, Austria; 2 Department of Biology, Institute for Microbiology, Friedrich Alexander University Erlangen/Nuremberg, Erlangen, Germany

## Abstract

Infections with the human pathogen *Helicobacter pylori* (*H*. *pylori*) can lead to severe gastric diseases ranging from chronic gastritis and ulceration to neoplastic changes in the stomach. Development and progress of *H*. *pylori*-associated disorders are determined by multifarious bacterial factors. Many of them interact directly with host cells or require specific receptors, while others enter the host cytoplasm to derail cellular functions. Several adhesins (e.g. BabA, SabA, AlpA/B, or OipA) establish close contact with the gastric epithelium as an important first step in persistent colonization. Soluble *H*. *pylori* factors (e.g. urease, VacA, or HtrA) have been suggested to alter cell survival and intercellular adhesions. Via a type IV secretion system (T4SS), *H*. *pylori* also translocates the effector cytotoxin-associated gene A (CagA) and peptidoglycan directly into the host cytoplasm, where cancer- and inflammation-associated signal transduction pathways can be deregulated. Through these manifold possibilities of interaction with host cells, *H*. *pylori* interferes with the complex signal transduction networks in its host and mediates a multi-step pathogenesis.

## Review

The interaction between pathogens and tissue- or organ-specific target cells in their host determines the establishment and development of infectious diseases. Therefore, pathogens must expose adapted, but specialized factors to overcome the host defense mechanisms at the tissue surface. In the digestive tract, the gastric mucosa is covered by a thick mucus layer protecting the epithelium from protein-lysing enzymes, gastric acid and finally chyme, which can also contain unwanted bacteria and pathogens. Forming this first effective barrier, epithelial cells show an apico-basolateral organization, which is primarily maintained by tight junctions, adherence junctions and a strictly regulated actin cytoskeleton [1,2]. Functional tight junctions are crucial for the maintenance of epithelial polarity and cell-to-cell adhesion, and form a paracellular barrier that precludes the free passage of molecules. Tight junctions are composed of several types of transmembrane proteins (e.g. occludin, claudins, junctional adhesion molecules [JAMs]) that bind to cytoplasmic peripheral proteins (e.g. zonula occludens [ZO] protein-1, -2 and −3, cingulin or multi-PDZ protein-1 [MUPP1]) and link the transmembrane proteins to the actin cytoskeleton. Adherence junctions mediate intercellular adhesions between neighboring cells, control the actin cytoskeleton and, therefore, exhibit anti-tumor properties. They consist of the transmembrane protein E-cadherin that bridges adjacent epithelial cells with the intracellular actin cytoskeleton. This involves a signaling complex composed of β-catenin, p120-catenin, α-catenin and epithelial protein lost in neoplasm (EPLIN), which is recruited to the intracellular domain of E-cadherin. These dynamic intercellular junctions are crucial for the integrity of the gastric epithelium and protect against intruding pathogens [1,2].

*Helicobacter pylori* (*H*. *pylori*) is a bacterial class-I carcinogen [3] that specifically colonizes the gastric epithelium of humans as a unique niche, where it can induce inflammatory disorders (e.g. ulceration, chronic gastritis, etc.) and malignant neoplastic diseases (mucosa-associated lymphoid tissue [MALT] lymphoma and gastric cancer) [4,5]. To resist the hostile environment in the stomach, *H*. *pylori* has developed highly sophisticated mechanisms to establish life-long infections in the stomach if not therapeutically eradicated. This is why it is considered as one of the most successful bacterial pathogens. *H*. *pylori* induces gastritis in all infected patients, but only a minority of approximately 10-15% suffers from clinical symptoms. The reason for the different responses to *H*. *pylori* is not clearly understood, but many reports point to individual genetic susceptibilities of the host to *H*. *pylori*-associated disorders. Accordingly, genetic polymorphisms associated with an elevated risk for gastric cancer have been identified in genes encoding interleukins (e.g. IL-1β), tumor necrosis factor (TNF), cyclooxygenase-2 (COX2), and other host factors [6,7]. Aside from host factors, *H*. *pylori* isolates harbor different patterns of genetic elements encoding for bacterial factors that are crucially involved in persistent colonization and pathogenesis. Some of these have already been defined as virulence factors [8], while others might serve as important niche and colonization determinants [9] or are still under investigation for their pathological relevance.

**Figure 1:**
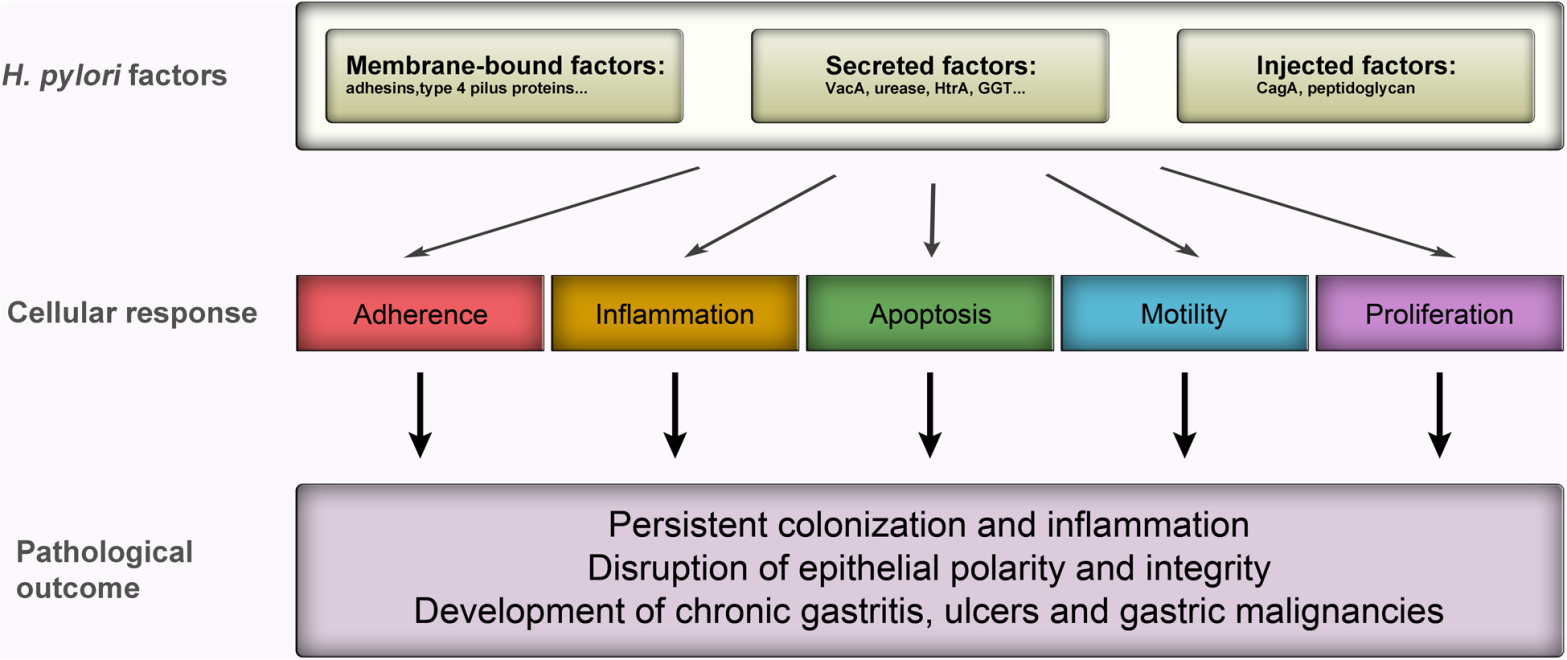
**Cellular responses to *H. pylori *upon colonization of a polarized epithelium.***H*. *pylori* expresses membrane-bound factors, secretes factors and exploits a type IV secretion system (T4SS) to inject effectors. These contribute to adhesion or induce signal transduction pathways leading to the induction of proinflammatory cytokine release, apoptosis, cell motility or proliferation. This network of diverse signaling pathways and cellular responses are involved in the establishment of persistent infection, inflammation and disruption of the epithelial polarity and integrity contributing to the development of gastritis, ulceration and gastric malignancies.

In the last three decades, remarkable progress has been made in the understanding of pathogenicity-related factors of *H*. *pylori* and their functional interaction with gastric epithelial cell components. These virulence-related factors are either secreted, membrane-associated, or translocated into the cytosol of host cells, where they can directly interfere with host cell functions (Figure 1). As a consequence of their different locations during the infection process, *H*. *pylori* is able to exploit a plurality of mechanisms to manipulate host cellular processes and to deregulate signaling cascades. The influence of *H*. *pylori* on these signaling pathways results in adherence, induction of proinflammatory responses through cytokine/chemokine release, apoptosis, proliferation, and a pronounced motogenic response as characterized *in vitro*. Taken together, these eventually result in persistent colonization, severe inflammation, disruption of the epithelial barrier function, and possibly gastric cancer (Figure 1). These effects originate from selective pathogen–host interactions, which have been summarized in this review to give a comprehensive overview of the large number of specialized bacterial factors and how *H*. *pylori* utilizes them to manipulate the gastric epithelium. Many of these factors act cooperatively, eventually leading to a complex scenario of pathogenesis-related signaling events.

### Membrane-associated factors: adhesins and beyond

**Figure 2:**
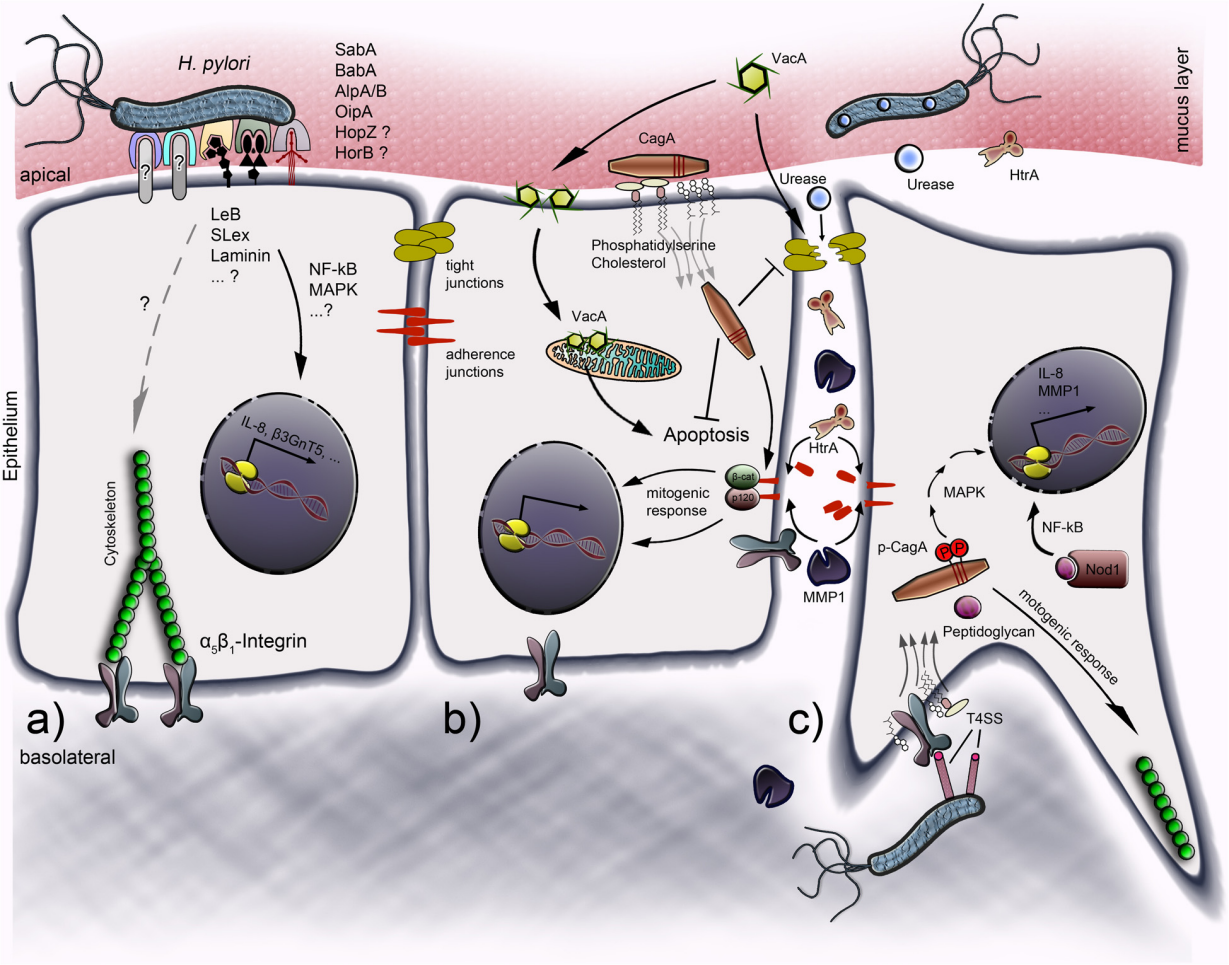
**Model of *H. pylori *factors interacting with host cells. (a)** At the apical side of the polarized epithelium *H*. *pylori* establishes the first adherence. SabA, BabA, AlpA/B, OipA, HopZ, HorB, etc. are considered as important adhesins that bind to host cell receptors (e.g. Le^b^, sLe^x^, laminin) and might contribute to NF-кB or MAPK signaling. **(b)***H*. *pylori* secretes VacA, which forms pores in the host membranes and localizes to mitochondria where it can interfere with apoptosis-related processes. Furthermore, VacA might influence the cellular barrier function by affecting tight junctions; an effect which has also been proposed for soluble urease. Together with *H*. *pylori*-secreted HtrA, which directly cleaves the adherence junction molecule E-cadherin, *H*. *pylori* efficiently disrupts the epithelial barrier. The T4SS injects the bacterial factor CagA. At the apical side of polarized cells, CagA might translocate via phosphatidylserine and cholesterol. In the cytosol of *H*. *pylori*-infected cells, CagA exhibits inhibitory effects on VacA-mediated apoptosis and the integrity of tight and adherence junctions. HtrA-triggered E-cadherin cleavage might be enhanced through *H*. *pylori*-induced MMPs and could increase the destabilization of the adherence complex composed of intracellular β-catenin and p120-catenin. Disruption of the E-cadherin complex might contribute to tumor-associated target gene expression in the nucleus and/or to the regulation of the actin cytoskeleton during cell morphological changes and motility. **(c)** Integrins are expressed at the basolateral side of a polarized epithelium and could be contacted by the T4SS adhesin CagL upon disruption of the intercellular adhesions. CagA translocates across α_5_β_1_-integrins and becomes rapidly tyrosine phosphorylated. Phosphorylated CagA then deregulates signal transduction pathways, leading to alterations in gene expression, and strongly interferes with the cytoskeletal rearrangement, which is important for the motogenic response to *H*. *pylori*. Peptidoglycan is considered to be another effector that binds Nod1, thereby activating the NF-кB signaling pathways.

Despite gastric peristalsis and transportation of chyme, *H*. *pylori* establishes a strong interaction with epithelial cells. In fact, adhesion of *H*. *pylori* is considered to be the first important step in pathogenesis in the stomach. The large group of outer membrane proteins (OMPs) contains some adhesins (e.g. blood-group-antigen-binding adhesin [BabA], sialic acid binding adhesin [SabA], adherence-associated lipoprotein A and B [AlpA/B], and outer inflammatory protein A [OipA]) that mediate binding of *H*. *pylori* to the host cell membrane, and other factors (e.g. lipopolysaccharide [LPS] and flagellin) that are able to trigger inflammatory responses in host tissues (Figure 2a).

Although bacterial adherence is crucially important for *H*. *pylori* pathogenesis, data showing direct effects of the above adherence factors on signaling pathways are scarce. This indicates that canonical adhesins may not directly activate signaling, but rather mediate a tight interaction between *H*. *pylori* and the host target cell, probably paving the way for additional bacterial factors to interact with their cognate receptors. In addition to OMPs and adhesins, flagellin and LPS have been widely investigated to address their role in *H*. *pylori* pathogenesis. In general, flagellin and LPS are important factors in many other bacterial infections, but it is unclear to what extent both factors contribute to *H*. *pylori*-induced signaling events. In contrast to the flagellin of other bacterial pathogens, *H*. *pylori* flagellin has only a very low capacity to stimulate toll-like receptor 5 (TLR5)-dependent interleukin-8 (IL-8) release [10]. This has been confirmed by the finding that purified *H*. *pylori* flagellin is a poor ligand for TLR5 [11]. Little information is available on the effects of *H*. *pylori* LPS on epithelial cells, indicating a yet undefined role in the *H*. *pylori*-infected epithelium as well. However, it has been suggested that *H*. *pylori* LPS might be a TLR2 agonist in gastric MKN45 cells, contributing to the activation of nuclear factor kappa B (NF-кB) and chemokine expression independently of the canonical LPS receptor TLR4 [12]. However, several factors have been well established as *H*. *pylori* adhesins that have the potential to alter signal transduction pathways, either by binding directly to cell surface receptors or acting indirectly, bringing other bacterial factors in a position to interact with cell surface structures which normally lack the capacity for signal transduction.

### Blood-group-antigen-binding adhesin (BabA)

*H*. *pylori* adhesion has been correlated with the presence of fucosylated blood group antigens [13] and the OMP BabA was subsequently identified as the first adhesin of *H*. *pylori* that binds to the fucosylated blood group 0 antigens Lewis B (Le^b^) and the related H1 on the epithelium [14]. However, the binding specificity of BabA to blood group 0 antigens is restricted to certain *H*. *pylori* strains, termed “specialist” strains, while BabA from “generalist” strains equally binds fucosylated blood group A antigens [15]. Recently, Globo H hexaglycosylceramide was suggested as an additional BabA binding partner that might play a role in the infection of non-secretor individuals [16]. Interestingly, specialist strains were found predominantly in South American countries, where the blood group 0 phenotype predominates in the local population. This adaptability in the binding specificity of BabA could be attributed to the loss of selective pressure on blood group A and B binding, rather than active selection of specialist strains, for binding affinities in specialist strains do not excel those of generalist strains [15]. The analysis of the genetic basis of BabA revealed two BabA loci (BabA1 and BabA2, of which BabA1 is not expressed [17]) and a closely related paralogous BabB locus [14]. It has been suggested that BabA expression is regulated via phase variation and recombination events with the BabB locus, as several studies have shown loss- and gain-of-function mutations *in vitro* and *in vivo*[14,18-20]. Additionally, the genetic configuration of the *bab* genes has been shown to correlate with preferential localization in the stomach and the BabA/B setting correlates with the highest risk for gastric cancer [21].

BabA-mediated adhesion of *H*. *pylori* to gastric epithelial cells might enhance CagA translocation and the induction of inflammation [22]. Furthermore, triple-positive clinical *H*. *pylori* isolates (BabA^+^, VacAs1^+^, CagA^+^) show greater colonization densities, elevated levels of gastric inflammation and a higher incidence of intestinal metaplasia in *H*. *pylori*-infected patients as compared to VacAs1^+^, CagA^+^ double-positive variants [23]. Epidemiologically, triple-positive strains are correlated with the highest incidence of ulceration and gastric cancer [24].

### Sialic acid-binding adhesin (SabA)

Independently of the adherence to fucosylated blood group antigens via BabA, *H*. *pylori* binds to sialic acid-modified glycosphingolipids, in particular sialyl-Lewis x/a (sLe^X^ and sLe^a^), via the bacterial adhesin SabA [25]. Interestingly, sLe^X^ is absent in the healthy non-inflamed gastric mucosa, and therefore SabA-mediated adhesion becomes a relevant factor in bacterial persistence after successful colonization and establishment of inflammatory processes in the stomach [25]. Accordingly, Marcos and colleagues [26] were able to show that *H*. *pylori*-induced inflammation leads to elevated expression of the glycosyltransferase β3GnT5, which acts as an important factor in the biosynthesis of the sLe^X^ antigen. The induction of β3GnT5 was dependent on tumor necrosis factor alpha (TNF-α), but not IL-8, and cells expressing ectopic β3GnT5 gave higher adhesion rates for SabA-positive *H*. *pylori* strains [26]. Like the situation with OipA and BabA, expression of SabA is subject to phase variation and gene conversion with its paralog SabB [27]. Additionally, acid-responsive signaling in *H*. *pylori* limits SabA transcription, which indicates that *H*. *pylori* adhesion is a dynamic and regulated process [28,29].

### Adherence-associated lipoprotein A and B (AlpA/B)

The OMPs AlpA and AlpB were initially described as proteins that facilitate binding *of H*. *pylori* to Kato-3 cells and the apical surface of gastric tissue sections [30,31]. AlpA and AlpB share a high degree of homology and are co-transcribed from the same operon. Moreover, both proteins are necessary for *H*. *pylori*-mediated adhesion to gastric biopsies [31]. In contrast to other adhesins, AlpA and AlpB are not subjected to phase variation and virtually all clinical isolates express both Alp proteins [32,33]. Importantly, deletion mutants lacking AlpA/B showed severe colonization defects in mouse and guinea pig animal models [33,34]. In sharp contrast, a recent study in Mongolian gerbils suggests that AlpA/B-deficient strains lead to exuberant gastric inflammation, as compared to the isogenic gerbil-adapted wildtype strain [35]. The reason for these conflicting results in different experimental settings remains unclear.

Interestingly, Lu et al. described significant differences in the activation of signaling pathways (mitogen-activated protein kinases [MAPKs], c-Fos, and c-Jun-, cAMP response element-binding protein [CREB]-, activator protein-1 [AP-1]-, and NF-κB-related signaling) induced by *H*. *pylori* AlpA/B deletion mutants [33]. These data imply that AlpA/B-mediated adherence facilitates a stronger activation of certain signal transduction pathways. However, injection and phosphorylation of CagA, as well as IL-8 induction, were not significantly affected by AlpA/B deletion [36]. *H*. *pylori* has been shown to bind components of the extracellular matrix (ECM), especially collagen IV and laminin [37], which have been proposed as candidate host factors acting as receptors. In this context, AlpA/B has been implicated in the adhesion to laminin [35]. As one of the major components of the ECM, laminin binds to integrin; hence, it would be interesting to investigate whether AlpA/B can indirectly modulate integrin signaling through binding to laminin.

### Outer inflammatory protein A (OipA)

OipA also belongs to the OMP group, and has been suggested to amplify IL-8 secretion via interferon-stimulated responsive element (ISRE) acting in parallel to the *cag*PAI-dependent mechanisms [38,39]. This is in contrast to other re-complementation studies indicating that OipA primarily functions in *H*. *pylori* adhesion to host cells, while the IL-8 level remains unaffected [36,40]. The reason for these opposing observations is not clear.

Yamaoka and co-workers have reported that the expression of functional OipA in *H*. *pylori* is phase-variable, and can be switched “on” or “off” by a slipped strand mispairing mechanism during chromosomal replication [39,41,42]. The OipA expression status is often associated with the presence of *cag*PAI, VacAs1, and VacAm1 allelic variants in western-type clinical isolates [40,43,44]. Therefore, it is difficult to provide relevant correlations between OipA status and clinical manifestation, for the OipA status does not seem to be completely independent of other disease-relevant genetic factors of the bacterium.

However, like other adhesins, OipA appears to be an important factor in the Mongolian gerbil infection model, since OipA-deficient strains failed to establish an infection and did not induce chronic inflammation and gastric metaplasia [45,46]. To date, no specific receptor or surface molecule for OipA binding has been described.

Nevertheless, based on infections with an *oipA* deletion mutant, OipA has been suggested to induce phosphorylation of focal adhesion kinase (FAK), leading to downstream activation of the MAPKs extracellular signal-regulated kinases 1 and 2 (Erk1/2) and the formation of actin stress fibers [47]. Collectively, these data indicate a host cell receptor with the capability of transmitting signal transduction in response to OipA; hence, it would be interesting to investigate whether recombinant OipA can bind to a host cell receptor and induce FAK signaling. As implied by a genomic knock-out mutant, OipA-mediated FAK activation might be a consequence of altered epidermal growth factor receptor (EGFR) signaling [47,48]. However, activation of EGFR has been convincingly shown to require a functional T4SS [49] and recombinant CagL alone is able to activate EGFR [50]. Additionally, an *oipA*-knock-out mutant of *H*. *pylori* was not able to trigger the EGFR signaling cascade involving phosphatidylinositide 3-kinases (PI3K) → phosphoinositide-dependent kinase-1 (PDK1) → Akt, which has been suggested to contribute to the regulation of FoxO forkhead transcription factor activity [51] and finally to the induction of IL-8 secretion [48]. In a recent study, it was proposed that EGFR/FAK/Akt signaling leads to phosphorylation of the focal adhesion protein paxillin, which then causes cytoskeletal reorganization and, subsequently, cell elongation [52].

In summary, OipA is an interesting *H*. *pylori* adhesion factor since it possibly interferes directly with signal transduction pathways that are predominantly activated by T4SS/CagA factors. This might indicate that OipA contributes to T4SS-dependent cellular responses, either through the direct activation of a yet unidentified receptor or indirectly through mediating tight adhesion between *H*. *pylori* and the host cell, leading to stronger T4SS/CagA-mediated signaling. In this context, it would be interesting to investigate whether the available *oipA* mutants still express fully functional T4SS pili.

### Other putative adhesins

In addition to the well described group of adhesion molecules, several other factors have been implicated in *H*. *pylori* adhesion to the gastric mucosa. The phase-variable protein HopZ has been suggested to play a role in bacterial adhesion [53] and recent studies have been able to demonstrate a role in the early phase of colonization. Re-isolates from a healthy volunteer challenged with HopZ ‘off’ *H*. *pylori* showed a strong *in vivo* selection for the HopZ ‘on’ status [54]. Another report by Snelling and co-workers proposed an adhesion-related function for HorB [55]. As an additional OMP, HopQ might also have an influence on bacterial adhesion. In a subset of tested *H*. *pylori* strains, *hopQ* deletion increased *H*. *pylori* adherence to AGS cells and led to a hyperadherent phenotype and subsequently to increased CagA phosphorylation, while IL-8 induction was not affected [56]. Accordingly, HopQ significantly decreased CagA injection in co-infection experiments in gastric epithelial cells [57]. The question of whether HopQ interferes with the function of other adhesins in certain *H*. *pylori* strains is still to be answered. Hence, recent findings showing that a HopQ knock-out mutant in another *H*. *pylori* isolate did not affect bacterial adhesion are not necessarily contradictory. The expression of HopQ contributed to *cag*PAI-dependent signaling and CagA injection, as these could be restored through *hopQ* re-expression [58]. These data suggest that *H*. *pylori* adhesins might act in two ways, either in a cooperating or in a masking manner.

### *H*. *pylori*-secreted urease, VacA and HtrA: priming factors in pathogenesis?

Secreted factors exhibit a high potential since they can act at the very beginning of microbial infections without requiring direct contact or adhesion to the host cells. In secretome analyses of *H*. *pylori*, a wide range of secreted or extracellular factors has been identified [59-61]. Although most extracellular proteins from *H*. *pylori* remain largely uncharacterized, our knowledge of γ-glutamyl transpeptidase (GGT), *H*. *pylori* neutrophil-activating protein (HP-NAP), urease, vacuolating cytotoxin A (VacA), and high temperature requirement A (HtrA) is steadily increasing. For example, GGT has been identified in the soluble fraction of *H*. *pylori*[59], and has been shown to enhance colonization of mice [62]. Interestingly, recombinant GGT can induce apoptosis and cell cycle arrest in AGS cells [63,64], but the molecular mechanism has not yet been elucidated. HP-NAP is a chemotactic factor of *H*. *pylori* that mainly attracts and activates neutrophils [65]; however, it does not play a prominent role during interactions with epithelial cells. Moreover, various direct effects of urease, VacA, and HtrA on gastric epithelial cells have been described, including induction of apoptosis and weakened integrity of intercellular adhesions (Figure 2b).

### Urease

The urease complex has often been described as a surface-presented virulence factor of *H*. *pylori*. The primary function of the urease machinery is buffering the acidic pH by converting urea to CO_2_ and ammonia, which is required for neutralizing the gastric acid around the bacteria. It has long been assumed that urease is secreted or surface-localized and contributes significantly to *H*. *pylori*’*s* ability to colonize and persist in the stomach, since it is actually considered to be an acid-sensitive bacterium [66]. The importance of urease for successful colonization has been highlighted in several studies [66-68]; however, an individual report indicates that urease-negative *H*. *pylori* strains are still able to colonize Mongolian gerbils [69].

The various sequenced genomes of *H*. *pylori* contain a urease gene cluster, which consists of seven conserved genes (UreA–B and E–I). UreA and UreB represent the structural subunits of a Ni^2+^-dependent hexameric enzyme complex. UreE, UreF, UreG and UreH are accessory proteins involved in nickel incorporation and enzyme assembly. Together with arginase, UreI is responsible for a sustained supply of urea under acidic environmental conditions [70]. In contrast to the hypothesis of surface-localized urease, another current model assumes that the main urease activity resides in the bacterial cytoplasm [71].

Apart from its role in the successful colonization of *H*. *pylori*, urease might also indirectly interfere with host cell functions. Urease-dependent ammonia production contributes to the loss of tight junction integrity in the epithelium, as demonstrated by decreased trans-epithelial electric resistance (TEER) and enhanced occludin processing and internalization in *in vitro* cultures [72]. Apparently, disruption of the tight junction integrity was independent of VacA and CagA in these studies, which is in sharp contrast to previous reports [73,74]. The effect of urease on tight junctions has been confirmed by another report showing that *ureB* deletion abrogates *H*. *pylori*’*s* ability to disturb tight junctions as a CagA- or VacA-independent process. By regulating the myosin regulatory light chain kinase (MLCK) and Rho kinase, UreB expression seems to be required for phosphorylation of MLC [75]. Even if the detailed mechanism through which *H*. *pylori* urease activates this signaling pathway remains unclear, these data can explain how urease contributes to the inflammatory responses that accompany the disruption of the epithelial barrier.

### Vacuolating cytotoxin A (VacA)

First evidence for a secreted vacuole-inducing toxin was found in experiments using filtrated *H*. *pylori* broth culture in 1988 [76]. This toxin was later identified as VacA [77,78]. The cellular responses to VacA range from vacuolization and apoptosis to the inhibition of T cell functions [79,80]. Due to these diverse cellular responses, VacA is considered to be a multifunctional toxin. However, in recent years it has become increasingly clear that most effects are due to the anion-channel function of VacA in multiple subcellular compartments and different cell types. Within the gene sequence, diversity of the signal sequence (allele types s1 or s2), intermediate region (allele types i1 or i2) and mid-region (allele types m1 or m2) has been observed [81,82]. As a consequence of its mosaic gene structure, the VacA protein is very heterogeneous and exists in different variants with differing activities.

VacA is expressed as a 140 kDa protoxin with an N-terminal signal region, a central toxin-forming region of 88 kDa (p88), and a C-terminal autotransporter domain, which is required for secretion of the toxin [83]. Upon secretion, VacA is further processed into two subunits, termed VacA^p33^ and VacA^p55^ according to their respective molecular weight, which form membrane-spanning hexamers [84,85]. It has been proposed that the VacA^p55^ domain is primarily responsible for target cell binding [86], while vacuolization requires a minimal sequence composed of the entire VacA^p33^ and the first ~100 amino acids of VacA^p55^[87,88].

The precise mechanism of VacA entry into target cells is still divisive, reflected by the fact that several putative receptors have been described. Presented on epithelial cells, EGFR might serve as a potential candidate to bind VacA prior to its internalization [89,90]. Further, receptor protein tyrosine phosphatases RPTPα [91] and RPTPβ [92] have been described as VacA receptors that promote VacA-dependent vacuolization. VacA binding to sphingomyelin in lipid rafts has also been shown to be an important event in VacA-mediated vacuolization [93]. In contrast to the induction of large vacuoles, VacA also promotes the formation of autophagosomes in gastric epithelial cells, which requires its channel-forming activity [94]. The low-density lipoprotein receptor-related protein-1 (LRP1) has been proposed to act as a receptor that interacts with VacA to promote autophagy and apoptosis [95]. Further putative host cell receptors for *H*. *pylori* VacA have been suggested; however, it remains uncertain whether they function as genuine receptors. Since it is not clear whether identified VacA receptors function independently of each other, the identification of such a diverse range of receptors implies a complex network of interactions and could explain the pleiotropic functions assigned to *H*. *pylori* VacA. In line with this assumption, purified and acid-activated VacA affected the transepithelial electrical resistance (TEER) of polarized epithelial cells [74], which is considered to be a strong indicator for the integrity of a polarized epithelial barrier. However, it is not known if this cellular phenotype requires a VacA receptor, although these reports indicate that VacA can exert very early effects during the multi-step infection by opening tight junctions and, consequentially, disrupting the epithelial barrier function.

It is well established that VacA is internalized and forms pores in membranes. This leads to an immense swelling, which consequently results in a vacuole-like phenotype of those organelles which harbor markers for both early and late endosomes [80]. In transfection experiments, the major consequence of VacA intoxication in gastric epithelial cells is clearly the induction of apoptosis in a mitochondria-dependent fashion [80]. A special hydrophobic N-terminal signal in VacA^p33^ subunit was identified in biochemical experiments that targets VacA to the inner mitochondrial membrane, where it also forms anion channels [96,97]. However, the precise route of VacA trafficking from endosomes to the inner membrane of mitochondria is still unknown. A recent study has suggested an important role for the pro-apoptotic multi-domain proteins BAX and BAK (both members of the Bcl-2 family) in membrane trafficking after vacuolization [98]. In this study, it was shown that translocation of *H*. *pylori* VacA to mitochondria and the induction of apoptosis strongly depends on the channel function of VacA. This leads to recruitment of BAX and, in turn, close contact of the vacuoles and mitochondria, and consequently, to co-purification of otherwise compartment-restricted marker proteins [98]. From genomic VacA-deletion and re-complementation analyses, Jain and colleagues concluded that the induction of apoptosis is preceded by a dynamin-related protein 1 (Drp1)-dependent mitochondrial fission and BAX recruiting and activation [99]. In conclusion, VacA intoxication can severely interfere with membrane trafficking and consequently disintegrate mitochondrial stability, which finally leads to cytochrome C release and apoptosis [80]. In previous studies, the anion-channel function of VacA was suggested to disrupt the inner membrane potential of isolated mitochondria [100], yet in the light of these recent studies, it is questionable whether VacA-induced loss of membrane potential is key in the apoptosis-inducing process of cytochrome C release.

### High temperature requirement A (HtrA)

In *Escherichia coli*, HtrA is a well-studied periplasmic chaperone and serine protease, and it has often been described as a bacterial factor contributing to the pathogenesis of a wide range of bacteria by increasing the viability of microbes under stress conditions [101]. Secretion of *H*. *pylori* HtrA was detected more than 10 years ago in comprehensive secretome analyses [60,61]. In fact, *H*. *pylori* HtrA is highly stable under extreme acidic stress conditions, suggesting that it could contribute to the establishment of persistent infection *in vivo*[102]. Like HtrA proteases from other Gram-negative bacteria, *H*. *pylori* HtrA contains an N-terminal signal peptide, a serine protease domain with a highly conserved catalytic triad, and two PDZ (postsynaptic density protein [PSD95], *Drosophila* disc large tumor suppressor [Dlg1], and zonula occludens-1 protein [ZO-1]) domains. Although its extracellular localization has been determined, it was unknown for long time whether HtrA exhibits a functional role in *H*. *pylori* infections. The investigation of *H*. *pylori* HtrA function is limited by the fact that all attempts to create a deletion or a protease-inactive *htra* mutant in the genome of *H*. *pylori* have hitherto failed [103,104].

Recently, a completely novel aspect of HtrA function has been discovered. It has been demonstrated that *H*. *pylori* HtrA is secreted into the extracellular space as an active serine protease [105] where it cleaves off the extracellular domain of the cell adhesion molecule and tumor suppressor E-cadherin [104]. Whether HtrA-mediated E-cadherin cleavage has an influence on the integrity and tumor-suppressive function of the intracellular E-cadherin signaling complex composed of β-catenin and p120 catenin is not yet known. Together with *H*. *pylori*-activated matrix-metalloproteases (MMPs) of the host cell [104,106], several modes of shedding and modifying cell surface molecules are now known. Mechanistically, E-cadherin ectodomain shedding leads to a local disruption of adherence junctions of polarized gastric epithelial cells which allows bacterial entry into the intercellular space [104]. This is supported by the observation that intercellular *H*. *pylori* could actually be detected in biopsies of gastric cancer patients [107].

The ability of purified HtrA to cleave E-cadherin *in vitro* and on gastric epithelial cells has also been demonstrated for other pathogens of the gastro-intestinal tract, such as enteropathogenic *E*. *coli* (EPEC) [108], *Shigella flexneri*[108] and *Campylobacter pylori*[108,109], but not for the urogenital pathogen *Neisseria gonorrhoeae*[108]. This indicates that HtrA-mediated E-cadherin cleavage is not unique to *H*. *pylori*, but might represent a more general mechanism to promote bacterial pathogenesis via bona fide virulence factors that requires transmigration across a polarized epithelium. The finding that HtrA cleaves E-cadherin supports the hypothesis that bacterial HtrA does not only indirectly influence microbial pathogenicity through improvement of bacterial fitness under stress conditions, but also exhibits direct effects on infected host cells.

### The *cag*PAI type IV secretion system and effectors

Another group of *H*. *pylori* factors is translocated into the host cell cytoplasm via a type four secretion system (T4SS). As effectors, cytotoxin-associated gene A (CagA) and peptidoglycan have been described to alter and/or trigger host cell signaling. While CagA may primarily function in the regulation of cell morphology and polarity [110,111], peptidoglycan has been described as a possible factor inducing nucleotide-binding oligomerization domain protein 1 (Nod1)-mediated NF-κB signaling (Figure 2c) [112,113]. However, there are other models for the role of Nod1 in *H*. *pylori* infection [114].

The T4SS is encoded by the cag pathogenicity island (*cag*PAI), which carries—depending on the clinical isolate—about 30 genes encoding for proteins that are necessary for pilus formation and T4SS function. The known structural and functional aspects of the T4SS have been summarized in several excellent reviews [115-117]. The current model of the T4SS involves structural core components forming a needle-shaped protrusion, which facilitates interaction with host-cell surface receptors and is indispensable for effector translocation into the host cell [115-117]. A comprehensive knockout study of all individual *cag*PAI genes by Fischer et al. defined an essential *cag*PAI-encoded protein repertoire that is required for CagA translocation, and in addition, an overlapping, but different panel of proteins that is required for IL-8 induction [118]. To date, the detailed mechanism of CagA transmembrane transport remains unclear; nevertheless, several host cell interactions with T4SS pilus proteins have been characterized, as discussed below.

### Interaction of the T4SS pilus with the cell membrane

In several *in vitro* studies, the interaction with β_1_-integrin has proven to be essential for CagA translocation [119,120]. The first and best characterized T4SS-dependent host cell interaction occurs between CagL and the α_5_β_1_-integrin on gastric epithelial cells [120]. CagL is localized on the surface of T4SS pili and serves as an adhesin crucial for CagA translocation, phosphorylation and IL-8 induction [118,120]. CagL harbors the classical integrin-activating Arg-Gly-Asp (RGD) motif, which is also found in natural integrin ligands like fibronectin or vitronectin [120,121]. It has been suggested that CagL binding to β_1_-integrin leads to the activation of β_1_-integrin and, subsequently, to activation of several host kinases, including FAK, Src, EGFR and HER3 (heregulin receptor 3)/ErbB3 in an RGD-dependent manner [50,120]. However, regulation of these signal transduction cascades might be more complex, since it has recently been proposed that a CagL/β_5_-integrin/ILK (integrin-linked kinase) stimulates EGFR → Raf → MAPK pathways independently of the RGD motif. In the same study, a weak interaction of CagL with the integrin β_3_-subunit was also observed, although no biological function has so far been described [122].

CagL binding to β_1_-integrin is necessary for the translocation of CagA [120]. In line with this, several other structural components of the T4SS pilus have been shown to bind to the β_1_-integrin subunit in Yeast-Two-Hybrid studies. These include CagI, CagY, and the translocated CagA itself, which are all thought to localize preferentially to the pilus surface and tip [119,123].

Considering the *in vivo* localization of the α_5_β_1_-integrin at the basal side of the epithelium, which is not accessible prior to the disruption of the epithelial integrity, the idea of an omnipresent CagA injection was highly appealing. Murata-Kamiya and co-workers observed that CagA binding to phosphatidylserine is a prerequisite for CagA translocation across the apical membrane [124]. In addition, cholesterol also appears to be a crucial membrane component for CagA transport. Several studies indicate that *H*. *pylori* targets cholesterol-rich lipid rafts [125], and cholesterol depletion impairs CagA translocation [126]. Of note, lipid rafts also harbor the α_V_β_5_ integrin complex [127]. However, no study has yet investigated the interplay of these putative entry mechanisms. Hence, it is conceivable that the above-mentioned molecules act in a cooperative fashion.

Another idea is that CagA is mainly translocated across the basolateral membrane of polarized cells, which is supported by the detection of tyrosine-phosphorylated CagA (CagA^pY^) in basolaterally expressed β_1_-integrin-based focal adhesions [120]. These represent hotspots of tyrosine phosphorylation events in cultured cells, which are important for CagA^pY^-dependent processes. In this context, the finding that the soluble *H*. *pylori* factors urease, VacA and finally HtrA can open tight junctions and adherence junctions supports this hypothesis, because *H*. *pylori* thereby directly disintegrates the polarized epithelium allowing direct contact between CagL and β_1_-integrin at the basolateral membrane of epithelial cells (Figure 2c).

### Role of intracellular CagA in eukaryotic signaling

CagA is one of the most abundant *H*. *pylori* proteins and has been found to be translocated into several gastric and non-gastric cell lines upon infection (listed in: [110]). Once inside the cell, CagA becomes rapidly tyrosine phosphorylated in its C-terminally located Glu-Pro-Ile-Tyr-Ala (EPIYA) motifs by host cell kinases [128-131]. CagL-β_1_-integrin interaction is required for CagA translocation; hence, tyrosine-phosphorylated CagA^pY^ is mainly localized in focal adhesions of cultured gastric epithelial cells along with CagA-phosphorylating kinases [120,130]. CagA^pY^ exhibits pronounced effects on the cell morphology of gastric epithelial cells [132,133], which putatively contribute to the disruption of the epithelial barrier *in vivo*. Depending on their surrounding sequence, the EPIYA motifs can be classified as EPIYA-A, EPIYA-B, EPIYA-C and EPIYA-D motifs. In western *H*. *pylori* strains, EPIYA-A, EPIYA-B, and varying numbers of EPIYA-C motifs have been found, whereas the combination of EPIYA-A and EPIYA-B with EPIYA-D motifs has been predominantly identified in East-Asian *H*. *pylori* isolates [134]. All types of EPIYA motifs can be phosphorylated, but not more than two simultaneously. Phosphorylation of EPIYA-C or EPIYA-D clearly primes phosphorylation of EPIYA-A or EPIYA-B, indicating a strict regulation of EPIYA motif phosphorylation, similar to what we know of tyrosine phosphorylation of mammalian factors [135]. Among the Src family kinases (SFKs), c-Src, Fyn, Lyn and Yes have been shown to phosphorylate CagA [128,129]. Recently, it was found that SFKs target the EPIYA-C/D motif, but not EPIYA-A or EPIYA-B [135].

SFKs and FAK become rapidly inactivated via a negative feed-back loop, which comprises binding of CagA^pY^ to SHP-2 and/or Csk (C-terminal Src kinase) [136-138]. The inactivation of SFKs then leads to the tyrosine dephosphorylation of ezrin, vinculin and cortactin, which are all important structural proteins in the regulation of the actin cytoskeleton [136,139,140]. Cortactin is also a substrate for Src, ERK, and PAK1, leading to a controlled phosphorylation pattern allowing regulated binding to FAK [141]. Although SFKs are inactive upon *H*. *pylori* infection, phosphorylation of CagA is maintained by c-Abl, which is obviously necessary for the functional activity of CagA in the cell morphological changes of cultured gastric epithelial cells [130,131]. In contrast to SFKs, c-Abl can target EPIYA-A, EPIYA-B and EPIYA-C motifs [135].

The way in which translocated CagA and/or CagA^pY^ interfere with host cell functions has not been fully investigated. The idea that bacterial CagA might function as a eukaryotic signaling adaptor upon translocation has arisen from observations of a transgenic Drosophila model. In the absence of the Drosophila Grb2-associated binder (Gab) homolog Daughter of Sevenless (DOS), CagA restored photoreceptor development, supporting the hypothesis that CagA can mimic the function of Gab [142]. To date, more than 25 proteins have been identified as possible interaction partners of CagA (Table 1), although it remains unclear which of them bind directly or indirectly (listed in [143]). CagA binds to a subset of proteins (Par proteins, c-Met, E-cadherin, p120 catenin, ZO-1, etc.) that are well known regulators of cellular polarity and adhesion independently of its tyrosine phosphorylation [143]. Accordingly, CagA might directly target intercellular adhesions by disrupting tight [73] and adherence junctions [144].

**Table 1:** **Overview of *H. pylori *factors that interfere with host cell functions**

	**Receptor / interaction partner**	**Described cellular responses / proposed protein functions**
Adhesins:		
BabA	Lewis B [14]; Lewis A [15]; Globo H hexaglycosylceramide [16]	Adhesion to host cells [14-16]
SabA	Sialyl Lewis X, sialyl Lewis A [25]	Adhesion to host cells [25], elevated binding via induction of β3GnT5 [26]
AlpA/B	Collagen IV, laminin [35,37]	Adhesion to ECM [35,37], reinforces NF-кB and MAPK signaling [33]
OipA	Not known	Adhesion to host cells [38-40], induction of inflammatory response [38,39]
HopZ	Not known	Adhesion?
HorB	Not known	Adhesion?
Secreted factors:		
Urease	Not known	Survival under acidic pH [66,70], disruption of tight junctions [72,75]
VacA	EGFR [89,90], RPTPα [91], RPTPβ [92], sphingomyelin [93], LRP1 [95]	Vacuolization [77,78], apoptosis [98,99], disruption of tight junctions [74]
HtrA	E-cadherin [104]	Disruption of adherence junctions [104]
GGT	Not known	Apoptosis [63], cell cycle arrest [64]
T4SS components:		
CagL	β_1_-Integrin [119,120]; (β_3_) β_5_-Integrin [122]	Facilitates CagA translocation [120]; activation of host kinases [120,122]
CagI	β_1_-Integrin [119,123]	Not known, necessary for CagA translocation and IL-8 induction [118,123]
CagY	β_1_-Integrin [119]	Not known, necessary for CagA translocation and IL-8 induction [118]
CagA	β_1_-Integrin [119]	Not known (for intracellular actions see below)
Injected factors:		
CagA	c-Met, p120, E-cadherin, Grb-2, Par proteins, PLC-γ, TAK, ZO-1, etc. [143]	Disruption of junctions and polarity, inflammation, proliferation [143]
Phospho-CagA	Src; SHP-2, Csk; c-Abl; Crk proteins, Grb2, Grb7, PI3K, Ras-GAP, SHP-1, etc. [143]	Cell elongation and cell motility [132,133,143], cancer development [146]
Peptidoglycan	Nod1 [113,152]	NF-κB activation [113]; AP-1 and MAPK activation [152]

On the other hand, CagA^pY^ interacts with many SH2 domain-containing signaling molecules (c-Abl, Src, Crk proteins, Grb proteins, Shp proteins, etc.), which are important for the regulation of proliferation, cell scattering and morphology. Remarkably, a selectivity of the EPIYA-A, EPIYA-B and EPIYA-C/D motifs in binding of downstream targets has been detected [145]. The *in vivo* importance of CagA phosphorylation is highlighted in transgenic mice studies demonstrating that CagA has oncogenic potential and can lead to the development of gastrointestinal and hematological malignancies. The occurrence of these phenotypes was dependent upon intact EPIYA motifs, as phosphorylation-resistant mutants failed to develop disease in the same experimental settings [146]. Hence, it is tempting to speculate whether it might be possible to employ selective SH2-containing peptides as selective inhibitors of distinct signal transduction pathways. In summary, CagA^pY^ and the regulated activities of SFKs and c-Abl control a network of downstream signal transduction pathways leading to morphological changes and motility of cultured gastric epithelial cells [111,147].

Interestingly, CagA and VacA functions antagonize each other in some experiments. VacA-induced apoptosis could be counteracted by both a phosphorylation-dependent and a phosphorylation-independent mechanism of injected CagA [148]. On the other hand, CagA-dependent cell elongation was decreased by VacA through inactivation of EGFR and HER2/Neu [149]. These studies underline the complex network of cellular effects which are induced by distinct bacterial factors.

### Peptidoglycan

In addition to their important functions in forming *H*. *pylori*’*s* cell shape and promoting colonization [150], peptidoglycans have also been described as *H*. *pylori* factors translocating into the cytoplasm of infected host cells where they bind to Nod1 in a T4SS-dependent manner [113]. Since it is well established that NF-κB activity is strictly T4SS-dependent, but CagA-independent [151], the finding of a T4SS-dependent intracellular peptidoglycan might add a piece to the puzzle of NF-κB regulation and could help to explain one possible upstream signal transduction pathway induced by *H*. *pylori*[112]. Nod1 might also influence the activity of AP-1 and MAPKs [152]. However, whether peptidoglycan prefers a T4SS-mediated translocation or transport across the membrane via outer membrane vesicles (OMVs) prior to NF-κB activation needs to be investigated in future studies [153].

## Conclusions

*H*. *pylori* expresses a large number of bacterial factors allowing interaction and interference with its host in multiple ways. This is reflected by the diversity of molecules that are either presented on the bacterial surface, shed/secreted or internalized into host cells. However, less is known about the local and/or time-phased interplay of these factors, which might act simultaneously or at different times in different cellular localities. Furthermore, factors have been studied that obviously have an impact on this multi-step pathogenesis, while their cellular function is not yet understood. Duodenal ulcer promoting gene A (DupA), for instance, represents a very interesting factor, since expression of DupA is considered as a marker for developing duodenal ulcer and a reduced risk for gastric atrophy and cancer [154]. It induces proinflammatory cytokine secretion by mononuclear cells [155], but the molecular mechanism is completely unclear. This is just one example indicating the strong interest in unraveling the molecular and cellular mechanisms through which pathogens modulate host cell functions, since they represent attractive targets for novel compounds in the selective fight against pathogens.

## Competing interests

The authors declare that they have no competing interests.

## Authors’ contributions

GP, SB and SW drafted and wrote the manuscript. All authors read and approved the final manuscript.
